# Intersubjectivity as an antidote to stress: Using dyadic active inference model of intersubjectivity to predict the efficacy of parenting interventions in reducing stress—through the lens of dependent origination in Buddhist Madhyamaka philosophy

**DOI:** 10.3389/fpsyg.2022.806755

**Published:** 2022-07-29

**Authors:** S. Shaun Ho, Yoshio Nakamura, Meroona Gopang, James E. Swain

**Affiliations:** ^1^Department of Psychiatry and Behavioral Health, Stony Brook University, Stony Brook, NY, United States; ^2^Pain Research Center, Division of Pain Medicine, Department of Anesthesiology, University of Utah School of Medicine, Salt Lake City, UT, United States; ^3^Program of Population Health and Clinical Outcomes Research, School of Public Health, Stony Brook University, Stony Brook, NY, United States; ^4^Department of Psychology, Stony Brook University, Stony Brook, NY, United States; ^5^Department of Obstetrics, Gynecology and Reproductive Medicine, Renaissance School of Medicine, Stony Brook University, Stony Brook, NY, United States; ^6^Department of Psychiatry and Psychology, University of Michigan, Ann Arbor, MI, United States

**Keywords:** intersubjectivity, parenting stress, relational worldview, free energy principle, parent-child dyadic interaction, dependent origination (pratītyasamutpāda), emptiness (Śūnyatā), maternal sensitivity

## Abstract

Intersubjectivity refers to one person’s awareness in relation to another person’s awareness. It is key to well-being and human development. From infancy to adulthood, human interactions ceaselessly contribute to the flourishing or impairment of intersubjectivity. In this work, we first describe intersubjectivity as a hallmark of quality dyadic processes. Then, using parent-child relationship as an example, we propose a dyadic active inference model to elucidate an inverse relation between stress and intersubjectivity. We postulate that impaired intersubjectivity is a manifestation of underlying problems of deficient relational benevolence, misattributing another person’s intentions (over-mentalizing), and neglecting the effects of one’s own actions on the other person (under-coupling). These problems can exacerbate stress due to excessive variational free energy in a person’s active inference engine when that person feels threatened and holds on to his/her invalid (mis)beliefs. In support of this dyadic model, we briefly describe relevant neuroimaging literature to elucidate brain networks underlying the effects of an intersubjectivity-oriented parenting intervention on parenting stress. Using the active inference dyadic model, we identified critical interventional strategies necessary to rectify these problems and hereby developed a coding system in reference to these strategies. In a theory-guided quantitative review, we used this coding system to code 35 clinical trials of parenting interventions published between 2016 and 2020, based on PubMed database, to predict their efficacy for reducing parenting stress. The results of this theory-guided analysis corroborated our hypothesis that parenting intervention can effectively reduce parenting stress if the intervention is designed to mitigate the problems of deficient relational benevolence, under-coupling, and over-mentalizing. We integrated our work with several dyadic concepts identified in the literature. Finally, inspired by Arya Nagarjuna’s Buddhist Madhyamaka Philosophy, we described abstract expressions of Dependent Origination as a relational worldview to reflect on the normality, impairment, and rehabilitation of intersubjectivity.

## Introduction

*The world is not an aggregation of things, but rather a symphony of relationships between many participants that are altered by the interaction*.

(Weber, 2017, p. 29)

An emerging view of evolution suggests that evolution of living systems is about survival-of-the-fitted—those entities that resist entropic destruction—rather than survival-of-the-fittest—the entities with the greatest reproductive success (Cohen and Marron, 2020). That is, survival requires a living entity to be integrated within biological and material networks to convert entropic disorganization into organization amid universal properties of energy, entropy, and interactions (Schrodinger, 2012; Friston, 2013; Ramstead et al., 2018). All biological substances, from a molecule to an organism, become what they are by interacting with something else in the environments (Gilbert et al., 2015) and they are impermanent as they become something different after each and every interaction with other objects (Weber, 2017). In short, living organisms are impermanent, inter-dependent, self-organizing systems in a universe of energy, entropy, and interactions.

As living systems are more appropriately considered as symbionts in symbiosis, as opposed to independent “individuals” existing in and of itself (Gilbert et al., 2012), human beings are no exception. The dyadic interactions between mother and infant constitute a prime example of inter dependence. Indeed, bidirectional moment-to-moment interactions between the symbionts, e.g., a mother and an infant, have long been recognized as important for infant development by developmental psychologists, e.g., (Bowlby, 1969; Stern, 1971; Sander, 1977; Tronick et al., 1978; Beebe and Lachmann, 1998). Recently, a systematic review has parsed the literature on mother-infant interactions in terms of nine dyadic concepts, namely, Mutuality, Reciprocity, Attunement, Contingency, Coordination, Matching, Mirroring, Reparation, and Synchrony (Provenzi et al., 2018), which will be described later. While these dyadic concepts are known to exert multiple effects on the developments of IQ, conduct, secure attachment, and stress regulation (Provenzi et al., 2018), they are not yet integrated in a formal theoretical framework (such as an active inference framework to be described in this paper), despite the well-known emphasis of dyadic interactions in many developmental theories, e.g., (Stern, 1971; Sander, 1977; Tronick et al., 1978; Beebe and Lachmann, 1998). Partly due to the lack of such integration, very little is known about the effects of engaging in dyadic interactions on maternal health and well-being, as acknowledged by Provenzi et al. (2018).

In this paper, we aim to address the gaps between these concepts in parent-child relations and a formal dyadic model that can provide heuristics for therapeutic interventions to promote the wellbeing of mother-child dyad. First, we postulate that intersubjectivity is a hallmark of quality dyadic interactions. Second, we introduce an active inference framework, namely, Free Energy Principle (FEP), to describe a person in a weakly coupled state and then propose our own dyadic active inference model to model dyadic interactions in a strongly coupled state, such that the causal link between intersubjectivity and maternal wellbeing (specifically, the reduction of parenting stress) is established. Third, we describe how maternal intersubjectivity can be impaired by problems of deficient relational benevolence, under-coupling, and over-mentalizing, with brain-based evidence for our theory. Fourth, to further corroborate our theory, we present a theory-driven literature review, using a coding system derived from our dyadic model to code recent clinically studied parenting interventions that measured parenting stress index (PSI; Abidin, 1995) as one of the outcome variables. Fifth, we integrate our work with the literature of dyadic concepts, using the meta-analytical review by Provenzi et al. (2018). Finally, inspired by Buddhist Madhyamaka Philosophy, championed by Arya Nagarjuna (ca 150–250 CE), we describe a relational worldview in terms of an abstract expression of Dependent Origination. By applying this abstract expression of Dependent Origination to the domains of physics, awareness, intersubjectivity, and active inference, we wish to elucidate a profound relation between intersubjectivity and wellbeing, i.e., intersubjectivity is anti-stress.

## Intersubjectivity is a hallmark of quality dyadic interactions and wellbeing

*The acid test of every epistemology is, when all is said and done, the intersubjective relationship*.

(Fuchs, 2017, p. 27)

Intersubjectivity—the relation between subjects—has been a key concept in phenomenology (Zahavi, 2018). When referring specifically to *the awareness of others’ awareness*, intersubjectivity is synonymous to some definitions of empathy in psychology (Preston and Hofelich, 2012; Zahavi and Overgaard, 2013). The wellbeing of the child can be influenced by the mother-child dyadic interactions, and the quality of these dyadic interactions is directly related to the capacity of maternal intersubjectivity (Leerkes et al., 2009), which is also known as parental sensitivity (Ainsworth et al., 1978; Bernard et al., 2013), parental empathic attunement (Rowe and MacIsaac, 2004), parental reflective functioning (Fonagy et al., 1991; Slade, 2005), and parental embodied mentalizing (Shai and Belsky, 2011). Poor quality in parent-child interactions can cause chronic stress in children and consequently resulting in multiple physical and mental health problems that surface later in life (Shonkoff et al., 2012). Not surprisingly, the capacity of maternal intersubjectivity is also related to maternal wellbeing. The capacity of intersubjectivity can be compromised in mothers suffering from interpersonal aggression (Dayton et al., 2016) and depressive mood disorders (Bernard et al., 2018), leaving these mothers at risk for excessive parenting stress, as parenting stress is inversely associated with parental intersubjectivity (Shai et al., 2017). Impaired parental intersubjectivity can adversely affect the bonding with the spouse as well (Nakić Radoš, 2021). Fortunately, the impairment of maternal intersubjectivity can be reversed. For example, we reported that a parenting intervention that increased the capacity of maternal intersubjectivity can reduce parenting stress with concomitant changes in the maternal brain regions that are known to mediate intersubjectivity (Ho et al., 2020).

The development of intersubjectivity in infants has been studied empirically since 1970’s. Among the pioneers, Colwyn Trevarthen and colleagues postulated the theory of “innate intersubjectivity” to account for the ontogeny of the active “self-and-other” awareness, stating that “*the infant is born with awareness specifically receptive to subjective states in other persons*,” and that a human being “*grows in active engagement with an environment of human factors – organic at first, then psychological or inter-mental*.” (Trevarthen, 1974; Trevarthen and Aitken, 2001). Trevarthen and Aitken (2001) suggested that intersubjectivity is as innate as *intrinsic motive formations* (IMFs) underlying three types of engagements with the world: (1) a “self-unity” that is innate and maintained by organismic self-organizing processes (IMFs) that regulate the physiological functions of the body to maintain a person’s self; (2) an agency that is developed to possess anticipatory control over the effects of actions and perceptions of objects in the environments; and (3) an inter-mental awareness (awareness of others’ purposes) that is developed through communications with other persons and dynamic interactive adjustments to others’ behaviors.

## Toward a dyadic model for intersubjectivity

Since infancy, we live our lives alternating between a weakly coupled state, in which we are not interacting with the environments, and a strongly coupled state, in which we are intimately interacting with others, e.g., moments of parent-infant interactions. In the science of complexity, the weakly coupled and strongly coupled states instantiate different phases of a complex system. In general, phase transitions produce discontinuity in the thermodynamic free energy of a complex system, such that a simple behavior in one phase may give rise to tremendous complexity in the other phase (Cocchi et al., 2017). Though the body of literature in complex systems is huge, here we only focus on the use of active inference framework to heuristically model a person in two phases separately—a weakly coupled state and a strongly coupled states—as follows.

### An active inference model in a weakly coupled state

The active inference framework is based on the premises that (1) perception and action of a person self-organize to minimize a quantity known as variational free energy and that (2) action selection, planning, and decision-making can be optimized by minimizing expected free energy, which quantifies the variational free energy of various actions based on expected future outcomes (Smith et al., 2022). Infants are born with self-unity that serves as a seed (ground zero) within innate complex self-organizing processes, as if they are objective perceivers and actors that are differentiated from other entities (Rochat, 2019). Such innate self-unity can serve as a seed (prior) in the active inference framework (Friston, 2018).

According to FEP, a living organism is a self-organizing system that maintains its characteristic phenotypic states and avoids surprising deviations from these expected states by generative processes that are self-organizing and self-evidencing. As the physical, biological processes of an organism embody its “best guess” about its environments, on average and over time the organism tends be attracted to a limited number of attractor states in the space of all possible states, with low entropy or spread in the probability density over the space of possible states, i.e., low variational free energy. Variational free energy is a measure of the upper bound of surprise or prediction error—the difference between the organism’s “best guess” beliefs about what caused its sensory states and what it observes (Friston, 2013; Ramstead et al., 2020; Friston et al., 2022).

Free energy principle adopts the notion of Markov blankets to define the boundary of the living system and its environments—which are partitioned as internal (systemic) states and external (environmental) states, respectively. The Markov blanket itself can be partitioned into active and sensory states, which can be differentiated as follows: active states are not influenced by external states, and sensory states are not influenced by internal states (Friston, 2013; Ramstead et al., 2018, 2020). The internal states and its Markov blanket together constitute an active inference engine that actively self-organizes to stay in the most probable expected states, i.e., the living system’s characteristic phenotypes.

Here we briefly describe the concept of Markov blanket as prescribed in FEP (Parr et al., 2022). Technically, a Markov blanket (*b*) is defined as follows:


p⁢(μ,x|b)=p⁢(μ|b)⁢p⁢(x|b)

This says that, statistically speaking, if *b* is known, then a variable μ is conditionally independent of a variable *x*. In other words, if knowing the values of *x* and μ both depends on the condition of knowing the value of *b*, then knowing *x* would give us no additional information about μ. To identify a Markov blanket in a system with conditional dependence, one can follow a rule that the blanket for a given variable comprises its *parents* (the variables it depends on), its *children* (the variables that depend on it) and, in some settings, the other *parents of its children*.

The FEP leverages the principle of minimizing variational free energy—the upper bound of surprise or prediction errors—to optimize the prior beliefs in the active inference engine. There are two ways to minimize variational free energy, i.e., perceptual inference and active inference. In perceptual inference, agents strive to update their prior beliefs, while in active inference agents change their environment (or their sampling of information from the environment) by selecting a plan or policy in a set of prior beliefs that would yield the least expected free energy (Peters et al., 2017). Notably, in FEP, the variational free energy is more of a function of beliefs and expectations in the internal states rather than a function of the environments hidden from the internal states (Ramstead et al., 2020). In such processes, internal and active states’ dynamics are a function of, and only of, a variational free energy bound on surprise, and the belief optimization is implicitly done in the minimization of variational and expected free energy (Friston et al., 2022).

The notion of active inference emphasizes that actions solicit a sensory outcome that informs approximate posterior beliefs about external states of the world. Such generative process in FEP renders a living organism to be participatory, or enactive in soliciting and therefore co-creating its perception of the external states, which is very different from a representationalist process by which external states generate sensory states exclusively (Friston et al., 2022). Heuristically, one may consider that an active inference engine is actively self-evidencing what the world should be (known as an enactive account), rather than passively learning to represent what the world seems to be (known as a representationalist account)—a distinction that has been elaborated in the literature (Ramstead et al., 2020). How this distinction is related to the differences in two incompatible worldviews will be clarified later.

**FIGURE 1:**
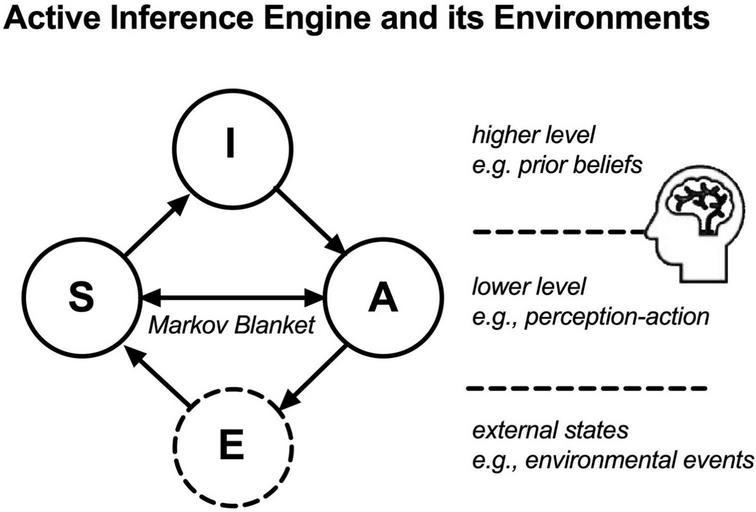
An active inference and its environments (external states): In an active inference model, an adaptive person functions as an active inference engine—consisting of nodes (A), (S), and (I; solid circles). In a hierarchical network, (S) represents the person’s afferent sensory state and (A) represents the person’s efferent active state, both at a lower level, and (I) represents the person’s prior beliefs at a higher level. Node (E) represents environmental events as external states (dashed circle). The bidirectional arrowed line between (A) and (S) indicates the notion of active inference, that actions solicit a sensory outcome that informs approximate posterior beliefs in the internal states (I) about the external states (E). This is done by minimizing variational free energy—the upper bound of surprise or prediction errors of the active inference. Nodes (E) and (I) do not have direct effects on one another, as they are separated by nodes (A) and (S) that serve as Markov blanket. Nodes (I) and (E) are statistically independent of each other given the Markov blanket, nodes (A) and (S). That is, the nodes (I) and (E) maintain a conditional independence of each other in the model, such that if the values of the Markov blanket nodes (S and A) are known, then knowing the internal states (I) does not provide any additional information about the external states (E), and *vice versa*. This conditional independence may give rise to the appearance of duality between the subject (the active inference engine) and the object (the external states) and is therefore considered a hallmark of a weakly coupled state of the active inference engine.

Inspired by FEP (Friston, 2013), we suggest that a person can be formally modeled as an active inference engine in a multi-level network consisting of four nodes, namely nodes of sensory states (S), active states (A), internal states (I), and external states or events (E). This network is partitioned into an external state (E) and an active inference engine that consists of the nodes (S) and (A) at a lower level and node (I) at a higher level. See Figure 1. The internal state (I) can be conceived as an innate prior—a set of “best guess” beliefs that may guide the active inference engine’s action planning and selection. When an event in the external states (E) interacts with a person, it can only affect internal states (I) indirectly through its interaction with the Markov blanket nodes (A) and (S), such that (E) afferently causes (S) to change and (A) efferently causes (E) to change; On the other hand, the nodes (S) and (A) at the lower level interact with the person’s prior beliefs in internal states (I) at the higher level, such that (S) causes (I) to change and (I) causes (A) to change.

In this model, nodes (A) and (S) are the Markov blanket of the node (E), because (A) is a *parent* of (E)—because (E) depends on (A)—and (S) is a *child* of (E)—because (S) depends on (E). In contrary, node (I) is not the Markov blanket of (E) because (I) is neither a *paren*t or *child* of (E), nor another parent of (E)’s child, (S). In this system, nodes (I) and (E) are conditionally independent of each other, under the condition of knowing the Markov blanket nodes (A) and (S). Conditioned on the Markov blanket, i.e., sensory states (S) and active states (A), the prior beliefs activated in internal states do not provide any additional information about the external state, (E), due to the conditional independence between nodes (I) and (E), as follows,


p⁢(I∩E|b)=p⁢(I|b)⁢p⁢(E|b)⁢wherein⁢b⁢refers⁢to⁢Markov

⁢blanket⁢nodes⁢(S)⁢and⁢(A)

Due to the conditional independence between nodes (I) and (E), the active inference engine and its external states are in a weakly coupled (conditionally independent) state, giving rise to the apparent duality between subject (the observer) and object (the observed), because knowing the former does not provide any information about the latter, and *vice versa*.

### A dyadic active inference model in a strongly coupled state

We need a dyadic model of two agents that are strongly coupled to model intersubjectivity that arises from subject-subject interactions. Just like ice and water are two phases of the same H_2_O molecules that behave distinctly (solid and liquid, respectively), the same active inference engine can behave very differently between the phases of weakly coupled and strongly coupled states—while an active inference engine maintains conditional independence between its internal and external states in a weakly coupled state, such conditional independence is diminished in a strongly coupled state, when its external states are no longer a unitary node (E), but rather another active inference engine, such that one engine’s active states (A) serve as a parent of the other engine’s sensory states (S), and *vice versa*. In the most strongly coupled state, one person’s active states will become total environmental inputs for the other person’s sensory states, and *vice versa*.

**FIGURE 2:**
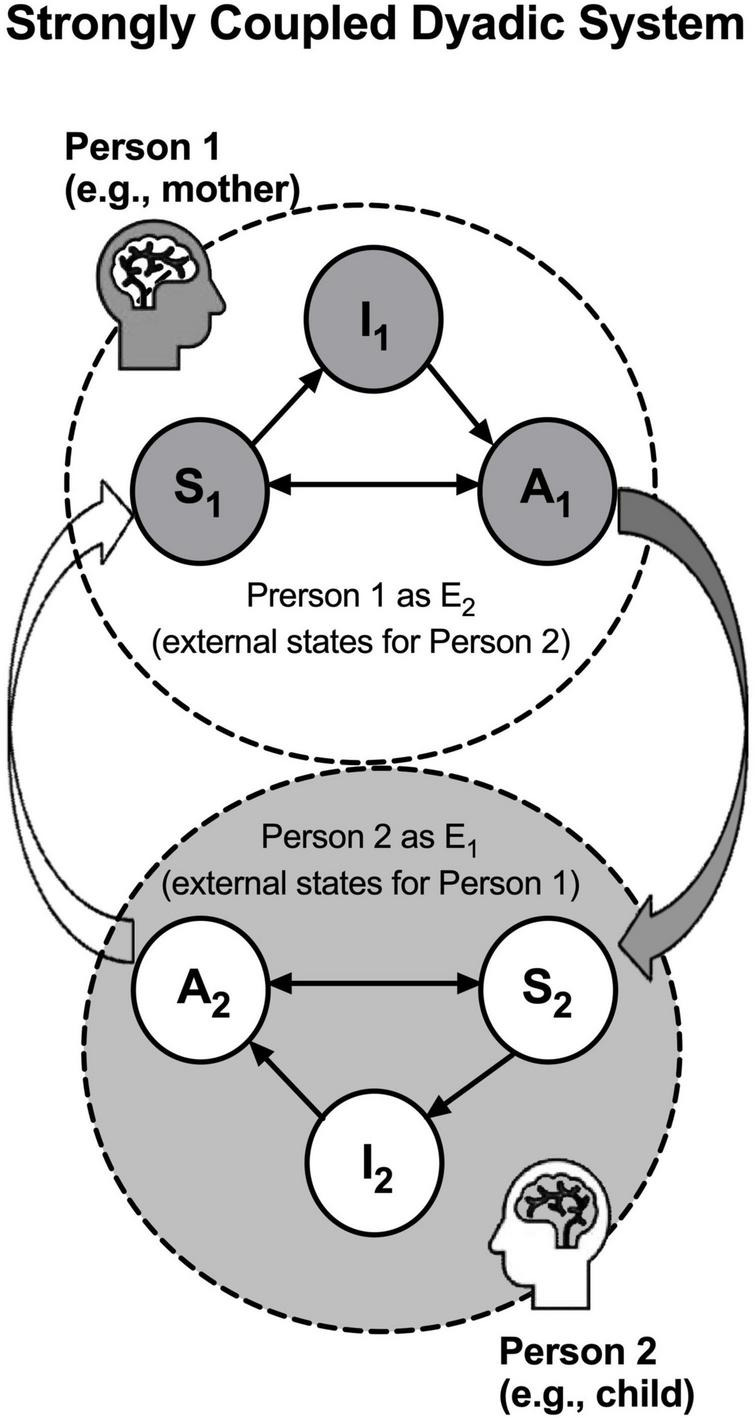
Active inference model in a strongly coupled state: When two persons (mother as Person 1 and child as Person 2) are strongly coupled, one person’s active states become the total environmental inputs for the other person’s sensory states, and *vice versa*. In this dyadic model, the strong coupling between the two persons is formed when their nodes (As) and (Ss) are coupled, wherein (A_1_) causes (S_2_) and (A_2_) causes (S_1_). Due to the strong coupling, the variational free energy in Persons 1 and 2 are also coupled and thus the prior beliefs in their internal states (I_1_ and I_2_) are optimized collectively. The two large, dashed circles indicate that there are no longer any unitary nodes (E) in the dyadic model, as the external states are now served by the multi-level network of the other person’s active inference engine.

Assuming this strongly coupled state in parent-child relationship, we have published a dyadic model to account for the inverse relationship between stress and intersubjectivity (Ho et al., 2020), wherein we studied how parental intersubjectivity is embodied and enacted in the brain. In this dyadic active inference model, when two active engines (two persons, say mother as Person 1 and child as Person 2) are strongly coupled, such that mother’s active state (A_M_) causes child’s sensory state (S_C_) and child’s active state (A_C_) causes mother’s sensory state (S_M_), one’s A and S become progressively similar to the other’s (A) and (S), respectively, over time, and prior beliefs in their internal states (Is) are hence attuned. Under such strong coupling, the two persons are actively inferring the other’s intentions in their prior beliefs hidden behind the sensory and active states and the variational free energy in the dyad is yoked as well. Thus, the strong coupling state mandates that one person’s prior beliefs about the other’s prior beliefs cannot reach the minimal variational free energy unless the other’s prior beliefs of the one’s prior beliefs also reach the minimal variational free energy—In other words, a higher level of intersubjectivity is attained if and only if the variational free energy in this strongly coupled state is minimized collectively. See Figure 2.

## The root of the impairment of intersubjectivity

The dyadic model of intersubjectivity that we proposed can explain why a child naturally wants to be mirrored and loved by the parent and will feel abandoned by an insensitive parent who neglects or dismisses this natural desire. Indeed, the relational benevolence—love and warmth for another person’s sake—in dyadic interactions has begun to be recognized to play a critical role in wellbeing (Maté, 2012). Unfortunately, not all parents are equal in their capacity of relational benevolence and intersubjectivity.

*But how come one can hold on to an invalid prior belief for so long, despite the excessive stress that renders his or her life miserable?*

Evidently, it is possible for humans to hold on to an outdated, invalid (mis)belief to the extent of becoming pathological, which is equivalent to keeping an overweighted prior in one’s active inference engine (Carhart-Harris and Friston, 2019). If a person fails to update or replace an outdated invalid belief, it may result in excessive variational free energy, and hence excessive stress (Peters et al., 2017; Goekoop and de Kleijn, 2021).

The misery of obsessively holding on to an invalid prior belief may be impossible for artificial intelligence (AI) programs—which can be considered as non-human inference engines (Friston et al., 2022)—because AI programs’ prior can be updated or replaced anew millions of times a day, without any stress or misery, such that they perform superbly, sometimes even outperform human champions, without any human assistance in games like chess, shougi, or go (Silver et al., 2017a,b).

We have postulated, in contrast to the AI programs, people suffer needlessly when they have invalid beliefs that do not reflect the reality, because invalid beliefs can cause human active inference engines to malfunction (Ho and Nakamura, 2017; Ho et al., 2021). The rehabilitation of impaired intersubjectivity is central to Indo-Tibetan Buddhist practices (Wallace, 2001). Informed by the central doctrines of Buddhism (Tenzin Gyatso, 1997, 2009), we specifically postulated (Ho et al., 2021) that the invalid beliefs that can cause the malfunctioning of active inference engines are called conceptual thoughts (*Vikalpas* in Sanskrit) that are laden with a non-relational view that there is a constant unchanging entity that is not changed by interactions and that an entity’s ultimate nature is identical to something observable (i.e., realism); and these invalid beliefs can be proliferated and embodied through processes called mental fabrication or superimposition (Prapañca in Sanskrit; Asanga, 2016).

In other words, we postulated that when a normal active inference engine is inflicted with non-relational prior beliefs (*Vikalpas*) in its internal states, which are invalid because they do not reflect the reality that all phenomena are products of subject-by-object interactions, the process of mental fabrication (Prapañca) will impair the active inference engine by holding on to invalid beliefs in the internal states, node (I), despite its failure to minimize variational free energy. We will discuss these Buddhist notions in the context of relational worldview to be presented later.

**FIGURE 3:**
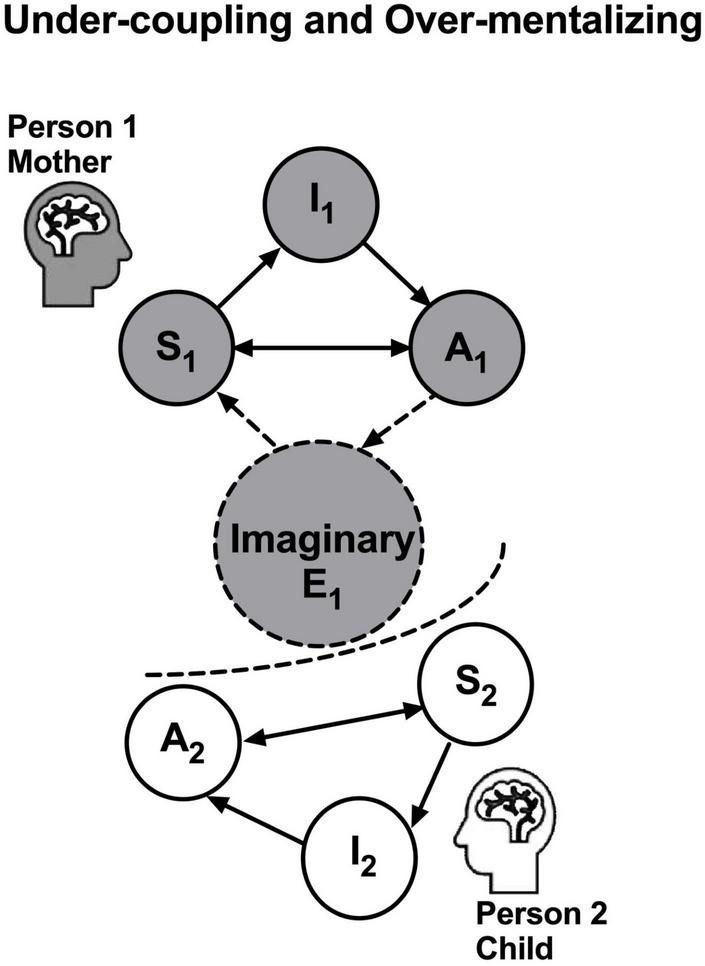
Under-coupling and over-mentalizing problems ensue in a dyadic system when Person 1 discards Person 2’s active inference engine and instead reduces Person 2 to an imaginary concept, namely Imaginary E_1_, as if it were a node E in a weakly coupled state, as denoted in the dashed circle in the center. Such imaginary E_1_ is therefore responsible for Person 1’s over-mentalization of Person 2. The dashed curve between Person 1 and Person 2 indicates the under-coupling, when Person 1 tends to ignore Person 2’s attempts to minimize variational free energy and instead treat Person 2 as an object in Person 1’s conceptual thoughts. The dashed arrows to and from Imaginary E_1_ indicate the lack of actual generative processes to minimize variational free energy in this pathological state.

In accordance with our postulation, we have theorized a dyadic model to explain the inverse relationship between parenting stress and maternal intersubjectivity and identified key brain regions that may mediate this relationship using a pre- and post-test design with the evidence-based “Mom Power” parenting intervention (Ho et al., 2020). In that report, we have identified three inter-related relational issues that may be addressed by dyadic interventions to reduce stress in dyadic interactions, namely, the problems of *(1) deficient relational benevolence due to invalid beliefs, (2) under-coupling, and (3) over-mentalizing*, as follows:

1. *Deficient relational benevolence:* Invalid beliefs prevents the awareness of relational benevolence, e.g., maternal empathic love and warmth toward the child’s internal states in the current context. When two persons (e.g., mother and child) are strongly coupled (**A_*mother*_ ≈ S_*child*_** and **A_*child*_ ≈ S_*mother*_**), the variational free energy are minimized collectively *if*, and *only if*, the prediction error in one person is minimized without increasing the other’s. Therefore, mother can achieve intersubjectivity by minimizing her variational free energy through communicative interactions with child, wherein mother’s prior belief would approximate child’s prior beliefs (**I_*mother*_** **≈ I_*child*_**). We have postulated that invalid beliefs (*Vikalpas*) will obscure the awareness of interdependence, and hence may diminish the awareness of relational benevolence and of the prior beliefs of each person’s active inference engine (Ho et al., 2021).
2. *Problem of under-coupling:* Under-coupling increases variational free energy. As depicted in Figure 3, when Person 1 engages Person 2’s overt behaviors only, Person 1 may reduce Person 2, who serves as Person 1’s external states, to a unitary object without its own inner states such as feelings and prior beliefs. Thus Person 1 would fail to achieve intersubjectivity and find it difficult to reduce stress in either party. For example, when mother neglects to see that her harsh reactions cause the child to feel negatively and only focuses on how to change child’s behaviors, mother would fail to recognize child’s attempts to reduce child’s own variational free energy and therefore mother’s variational free energy during dyadic interactions would increase. Being ignored or rejected, child’s stress (excessive variational free energy) would increase, which would, in return, increase mother’s stress.

3. *Problem of over-mentalizing*: Over-mentalizing can perpetuate impairments of intersubjectivity and exacerbate dyadic stress. When there is a disagreement or conflict between two persons, dyadic stress may increase if person 1 becomes defensive against person 2, as if person 2 were an enemy, and therefore misattributing person 2’s disagreeing behaviors to malice or character flaw, i.e., over-mentalizing. For example, mother may over-mentalize child’s behaviors as “he does not respect me.” When mother’s over-mentalizing explains away child’s actual prior belief, she will not even recognize her own ignorance of child’s feelings and prior beliefs. Thus, when stress potentiates mother’s over-mentalizing, child’s disagreeing behaviors would only confirm mother’s preconceived existing biases against child, perpetuating the impairment of intersubjectivity in a vicious cycle. As described later, conceptual thoughts are responsible for the problem of over-mentalizing. As depicted in Figure 3, Imaginary E_1_ (in a dashed circle) denotes Person 1’s conceptual thoughts that may result in Person 1’s over-mentalization of Person 2. The dashed curve between Person 1 and Person 2 indicates the under-coupling, when Person 1 tends to ignore Person 2’s attempts to minimize variational free energy and instead treat Person 2 as an imaginary object in Person 1’s conceptual thoughts. The dashed arrows to and from Imaginary E_1_ indicate the lack of actual generative processes to minimize variational free energy in this pathological state.

## A brain model for intersubjectivity as a therapeutic target of parenting intervention

There is now preliminary neuroimaging support for brain networks that may mediate the effects of a parenting intervention on maternal intersubjectivity (Ho et al., 2021). In this study, we assigned mothers to “Mom Power” intervention or a control condition and all of them underwent a child face mirroring task (CFMT), at pre-treatment and post-treatment (Ho et al., 2020). The CFMT was designed to elicit maternal intersubjectivity-dependent responses to their own children or unknown other’s children by asking the participants to imitate children’s emotional facial expressions or a control condition (simply observe without imitating), because voluntary imitation of others’ facial expressions is key to the development of intersubjectivity (Meltzoff and Moore, 1977). The results showed that the within-subject changes in parenting stress were positively associated with the concurrent changes in the differential responses during prediction error-related (positive vs. negative valence) contrast in the imitating (mirroring) own child’s faces vs. its control condition in the periaqueductal gray (PAG), a subcortical region related to fight-or-flight defensive motivation, and, conversely, negatively associated with those in the amygdala and nucleus accumbens (NAc), two subcortical regions related to social reward motivation. Moreover, the within-subject changes in parenting stress were positively associated with the functional connectivity between the dorsomedial prefrontal cortex (dmPFC) and PAG, and, conversely, negatively associated with concurrent changes in the functional connectivity between dmPFC and NAc, during the imitating (mirroring) own child’s faces vs. its control condition. Connectivity with the dmPFC may be interpreted in relation to at least two functions: (1) social mirroring behaviors and (2) representing the significant other (Ho and Nakamura, 2017). The parenting intervention effects on stress reduction were partially mediated by differential changes in subcortical functional connectivity in maternal brain regions of NAc and PAG, which have also, respectively, been associated with maternal care vs. defense, respectively, (Numan, 2007; Swain et al., 2018). Notably, brain regions underlying surprise or deviation from expectation largely overlap with these subcortical motivational neurocircuits, including the amygdala, NAc, and PAG (Swain and Ho, 2017). Additionally, this model has been important in the interpretation of the differential effects of opioids on the maternal brain, which include disrupted connectivity between NAc and PAG (Swain and Ho, 2021).

The provisional success in identifying a brain model to support the dyadic active inference model encouraged us to conduct the following theory-guided analysis of published intervention studies.

## A theory-guided quantitative analysis of parenting intervention studies in the literature

*When the rose is gone and the garden faded you will no longer hear the nightingale’s song. The Beloved is all; the lover just a veil. The Beloved is living; the lover a dead thing. If love withholds its strengthening care*, *the lover is left like a bird without care*, *the lover is left like a bird without wings. How will I be awake and aware if the light of the Beloved is absent? Love wills that this Word be brought forth.*

Jalaluddin Rumi (Mathnawi I, 23–31)

Using Rumi’s poem as a metaphor, when primary caregivers are somehow laden with the problems of *deficient relational benevolence, under-coupling, or over-mentalizing*, the “garden” in which a child can thrive is faded, and the child is like a bird without wings. As described above, we postulate that parenting stress will mostly result from dysfunction of interaction processes associated with the three relational issues that we deduced based on the dyadic active inference model of intersubjectivity.

*Accordingly, we hypothesize that a parenting intervention should effectively reduce parenting stress if the intervention is designed to address these three issues by promoting relational benevolence and by training the skills to mitigate the under-coupling and over-mentalizing problems in parents.* To test this hypothesis, we conducted a theory-guided quantitative analysis of recently published studies of parenting intervention by developing a coding system to parse parenting interventions published between year 2016 and 2020 and examined whether results based on the coding were associated with the effects of parenting interventions, as compared to a control or baseline condition, on PSI, one of the most common measures of parenting stress (Abidin, 1995).

### Methods of the theory-guided quantitative analysis

We used PubMed database to search for randomized controlled trials (RCTs) reported in English in the last 5 years prior to January 14, 2021, using the following keywords: “Parenting intervention,” “RCT” or “randomized controlled trial,” and “PSI.” We found 52 studies that met inclusion criteria and screened out 17 of them due to the following reasons: (1) the lack of PSI total score as an outcome variable, (2) the absence of comparisons between a intervention condition and a control/baseline condition, or (3) the presence of a medical condition, e.g., traumatic brain injury in the child, that may originate from and/or result in complications in the social environments beyond the parent-child dyads. The list of the final 35 studies reviewed and the coding results for each study are presented in Table 1. These studies were coded by two authors SSH and MG (hereafter Raters 1 and 2, respectively) independently, according to the following binary coding scheme.

**TABLE 1:** The coding of studies included in the theory-guided quantitative analysis.

PMID	First author	Journal	Year	Target population	Sample size per group	Tx effect on PSI 1 = positive effect, 0 = otherwise	Component 1 1 = criteria met 0 = else	Component 2 1 = criteria met 0 = else	Component 3 1 = criteria met 0 = else
32817266	Medoff CB	Pediatrics	2020	Parents of infants who underwent surgery for congenital heart disease	Tx *n* = 71, Control *n* = 70	0	0	1	0
32432487	Cala Cala LF	Clin Pediatr (Phila)	2020	Low income new mothers	Tx *n* = 150, Control *n* = 150	1	1	0	1
32027150	Ross AM	J Fam Psychol	2020	Military families	Tx *n* = 53, Control *n* = 51	0	0	1	1
31808376	Whittemore R	Diabetes Educ	2020	Parents of youths w/Type 1 diabetes mellitus	Tx *n* = 81, Control *n* = 81	1	1	1	1
31583748	Poehlmann-Tynan J	Infant Ment Health J	2020	Parents of preschool children	Tx *n* = 25, Control *n* = 14	0	0	0	1
31342445	Rollins PR	J Autism Dev Disord	2019	Parents of children w/autism spectrum disorder	Tx *n* = 32, Control *n* = 24	1	1	1	1
31522896	Chen H	Patient Educ Couns	2020	Parents of children w/congenital cataract	Tx *n* = 93, Control *n* = 107	1	1	1	0
31107793	Knight RM	J Pediatr Gastroenterol Nutr	2019	Mothers of children w/behavioral feeding disorder	Tx *n* = 12, Control *n* = 12	1	1	1	1
31222789	McCarter DE	J Adv Nurs	2019	Mothers w/depression and anxiety symptom	Tx-I *n* = 181, Tx-II *n* = 189, Control *n* = 167	0	0	0	0
31165715	Sawyer A	J Med Internet Res	2019	New mothers w/depression and parenting problems	Tx *n* = 72, Control *n* = 61	0	1	0	0
31023190	O’Shea A	Psychiatr Serv	2019	Mothers w/schizophrenia spectrum or mood disorder	Tx *n* = 66, Control *n* = 65	1	1	1	1
30804992	Sgandurra G	Neural Plast	2019	Parents of low-risk preterm infants	Tx *n* = 24, Control *n* = 20	1	1	1	0
29855840	Lutenbacher M	Matern Child Health J	2018	Hispanic mothers of newborns	Tx *n* = 91, Control *n* = 83	1	1	1	0
29953626	Ericksen J	Infant Ment Health J	2018	Mothers w/a range of postnatal mental disorders, e.g., depression	Tx *n* = 16, Control *n* = 15	1	1	1	1
29921144	Luby JL	Am J Psychiatry	2018	Parents of children w/early developed depressive symptoms w/comorbidity of externalizing disorder.	Tx *n* = 115, Control *n* = 114	1	1	1	1
29413437	Kaltenbach K	Drug Alcohol Depend	2018	Mothers w/opioid use disorder	Tx *n* = 96 Pts in a within-subject design.	0	0	0	0
28929582	Hemdi A	Child Care Health Dev	2017	Mothers of chiildren w/autism spectrum disorder	Tx *n* = 34, Control *n* = 33	1	0	1	1
28881303	Lachman JM	Child Abuse Negl	2017	Parents of children at risk for maltreatment	Tx *n* = 34, Control *n* = 34	1	1	1	1
28830853	Boogerd E	J Med Internet Res	2017	Parents of child w/type 1 diabetes	Tx *n* = 54, Control *n* = 51	0	0	0	0
28739559	Sawyer MG	J Med Internet Res	2017	New mothers	Tx *n* = 491, Control *n* = 328	0	0*	0*	0
28647759	Rosenblum KL	Arch Womens Ment Health	2017	Mothers w/at least one of the following conditions: 1. a mother’s history of childhood maltreatment, 2. adult interpersonal violence, 3. past or current depression and anxiety.	Tx *n* = 68, Control *n* = 54	1	1	1	1
28512921	Jones SH	J Child Psychol Psychiatry	2017	Parents w/bipolar disorder	Tx *n* = 47, Control *n* = 50	1	1	1	1
28464006	Koushede V	PLoS One	2017	Expectant mothers	Tx *n* = 863, Control *n* = 863	0	0	0	1
28410972	Luthar SS	Womens Health Issues	2017	Mothers w/work related burnout in medical settings	Tx *n* = 21, Control *n* = 19	1	1	1	1
27306883	Thijssen J	Child Psychiatry Hum Dev	2017	Parents of children w/ADHD	Tx *n* = 91, Control *n* = 55	1	1	1	1
27624608	Ehrensaft MK	J Prim Prev	2016	Mothers in college w/relatively high parental stress	Tx *n* = 26, Control *n* = 26	1	1	1	1
27878951	Hodes MW	J Appl Res Intellect Disabil	2017	Parents w/mild intellectual disabilities or borderline intellectual functioning	Tx *n* = 43, Control *n* = 42	1	1	1	1
27710006	DeVoe ER	Psychol Trauma	2017	Parents in military service about to be deployed	Tx *n* = 57, Control *n* = 58	1	1	1	1
27464071	Natrasony C	Phys Occup Ther Pediatr	2016	Mothers of children w/gross-motor delays	Tx *n* = 23, Control *n* = 16	0	0	1	0
27449367	Castel S	Early Hum Dev	2016	Parents of preterm infants	Tx *n* = 33, Control *n* = 32	1	1	1	1
26446726	Bagner DM	J Abnorm Child Psychol	2016	Mothers from underserved population	Tx *n* = 31, Control *n* = 29	0	1	1	0
27258925	Leung C	Res Dev Disabil	2016	Parents of preschool children w/developmental disabilities	Tx *n* = 62, Control *n* = 57	0	1	1	0
27302544	Ngai FW	J Psychosom Res	2016	Mothers w/postpartum depression	Tx *n* = 197, Control *n* = 200	1	0	0	1
26986919	Walton K	Can J Public Health	2016	Parents of preschool children	Tx *n* = 29, Control *n* = 25	1	0	1	1
26939716	Fonagy P	Infant Ment Health J	2016	Mothers at risk for mental health issues	Tx *n* = 38, Control *n* = 38	1	1	1	1

* Raters 1 and 2 differed in the coding.

1. To meet Component 1 (promotion of relational benevolence through enhancing awareness of the child’s internal states and the importance of love and warmth in dyadic interactions), a treatment (Tx) should have ALL of the following: 1. Specific own child in question; 2. Education on child’s social developmental needs, including the development of secure attachment in the child’s prior beliefs, which are only made possible through dyadic interactions; and 3. Emphasize the importance of the caregiver’s positive stance, e.g., warmth, love, sensitivity, etc.
2. To meet Component 2 (intervention to reduce under-coupling), a Tx should have ALL of the following: 1. Asking the parent to realistically observe the child’s behaviors, with sufficient consistency with what another observer would agree, i.e., valid observation; 2. Education of behavioral techniques contingent on actual feedback from the child’s response during parent-child interactions.
3. To meet Component 3 (intervention to reduce over-mentalizing), a Tx should have ALL of the following: 1. Skill training on how to observe one’s thoughts and feelings with non-judgmental stance, without necessarily reacting to thoughts and feelings, e.g., mindfulness; 2. Education on how one’s moods and beliefs may negatively influence one’s projection/mentalizing of others and may increase distress tolerance when parents are in negative moods, e.g., feeling frustration.

The Components 1–3 in the coding scheme corresponded to the three components of the dyadic active inference model, which we developed and presented above to address the problems of *(1) deficient relational benevolence, (2) under-coupling, and (3) over-mentalizing*, respectively. Notably, the major distinction between Component 1 and other Components is that Component 1 serves as a mindset, a frame centered in the dyad, not a single person, with an emphasis on unconditional positive regards, e.g., love and warmth, of the relation, while Components 2 and 3 are more specifically contingent upon specific situations and skill oriented. The major distinction between Components 2 and 3 is that Component 2 should be focused on child-oriented observations with a data-driven approach, not inward observations of parents’ internal working model of the child. Conversely, Component 3 should be focused on parental inward-observations of parental thoughts and emotions when they are used to mentalize the child.

The outcome variable, the Tx effect on PSI, was coded according to the following rule: If there was a statistical significant difference in PSI (total score) between the intervention (Tx) and Control groups, as a significant Group main effect or a Time-by-Group interaction effect, or a within-subject difference from a baseline, such that the PSI total score was lower in the Tx than the control condition, then the Tx effects of PSI was coded as “1” (positive effect), otherwise as “0” (negative effect).

### Results of the theory-guided quantitative analysis

#### Inter-rater reliability

The coding of Components 1–3 showed superb inter-rater reliability between the two raters. For Component 1, Rater 1 coded 23 studies as “1” and 12 studies as “0.” The two raters’ coding were identical for all 35 studies, except one study (#20), which Rater 1 and 2 coded as “0” and “1,” respectively. The inter-rater reliability for Component 1 was very high (measurement of agreement kappa = 0.935, asymptotic standard error = 0.064, approximate *T* = 5.545, with approximate significance, *p* < 0.001). For Component 2, Rater 1 coded 26 studies as “1” and 9 studies as “0.” The two raters’ coding were identical for all 35 studies, except one study (#20), which Rater 1 and 2 coded as “0” and “1,” respectively. The inter-rater reliability for Component 2 was very high (measurement of agreement kappa = 0.922, asymptotic standard error = 0.076, approximate *T* = 5.473, with approximate significance, *p* < 0.001). For Component 3, both Rater 1 and 2 coded 23 studies as “1” and 12 studies as “0.” The two raters’ coding were identical for all 35 studies. The inter-rater reliability for Component 3 was perfect (measurement of agreement kappa = 1.00, asymptotic standard error = 0.00, approximate *T* = 5.916, with approximate significance, *p* = 0.000). The inter-rater reliabilities for all Components were high, providing evidence for the reliability of coding of three Components for 35 studies. The two raters discussed the differences in coding and reached final agreements to use Rater 1’s coding in the following analyses.

#### Associations between the coding of Tx effects on parenting stress index and components 1–3

The non-parametric correlations (Kendall’s Tau-B and *p*-values) between the variables (the coding of intervention effects on PSI and Components 1–3) are summarized in Table 2. These results suggested that all three Components were significantly correlated with the intervention (Tx) effects on PSI total; Components 1 and 2 were highly correlated with each other; and Component 3 were not correlated with other Components, thus relatively distinct from either Component 1 or 2.

**TABLE 2:** The non-parametric correlations (Kendall’s Tau-B and *p*-values) between the variables.

	Tx effect on PSI	Component 1	Component 2	Component 3
Tx Effect on PSI	1			
Component 1	0.620 **	1		
	*p* < 0.001			
Component 2	0.539 **	0.539 **	1	
	*p* = 0.002	*p* = 0.002		
Component 3	0.620 **	0.239	0.264	1
	*p* < 0.001	*p* = 0.163	*p* = 0.124	

** p-value < 0.005.

The associations between each of the three Components and the coding of the outcome variable were independently tested using the directional association test, Sommer’s *d*. The results showed that each of the three Components can predict the Tx effect on PSI: For Component 1, the directional association, treating Component 1 as independent variable and the binary coding of Tx effects on PSI as dependent variable, was significant (Sommer’s *d* = 0.620, standard error = 0.143, *T* = 3.890, *p* < 0.001). For Component 2, the directional association, treating Component 2 as independent variable and the Tx effects on PSI as dependent variable, was significant (Sommer’s *d* = 0.585, standard error = 0.159, *T* = 3.033, *p* = 0.002). For Component 3, the directional association, treating Component 3 as independent variable and the Tx effects on PSI as dependent variable, was significant (Sommer’s *d* = 0.620, standard error = 0.143, *T* = 3.890, *p* < 0.001). The cross tabulations of the outcome variable (Tx effect on PSI) and each of the Components are summarized in Table 3. The inclusion of each Component in a particular intervention was found to be associated with the reduction of parenting stress. This supports the importance of each Component in interventions for parenting stress.

**TABLE 3:** The cross tabulations of the treatment effect on PSI and the coding of three components.

		Tx effect on PSI		
	Coding	Negative (Total # = 12)	Positive (Total # = 23)	Sum of row
Component 1	0	9	3	12
	1	3	20	23
Component 2	0	7	2	9
	1	5	21	26
Component 3	0	9	3	12
	1	3	20	23

**FIGURE 4:**
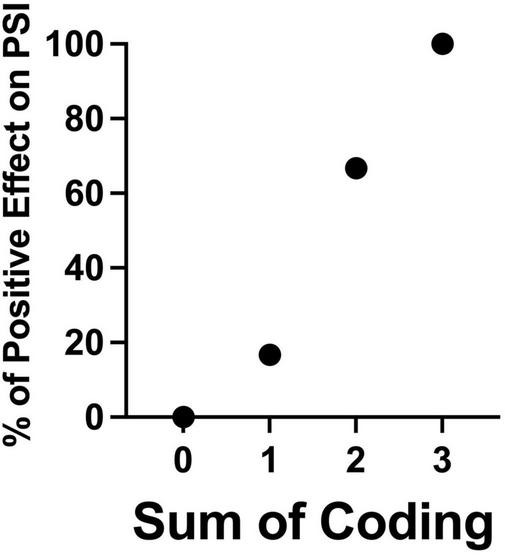
A monotonically increasing relation between the sum of coding of the studies included in the theory-guided quantitative analysis (on the *x*-axis) and the percentage of the included studies showing significant positive effects on reducing parenting stress (on the *y*-axis).

Additionally, to test the additive effects of Components 1, 2, and 3 on the outcome variable (Tx effect on PSI), we computed the sum of coding for each study (which yields a possible total value of 0, 1, 2, or 3). The directional association, treating the sum of coding as independent variable and PSI as dependent variable, was significant (Sommer’s *d* = 0.591, standard error = 0.064, *T* = 7.698, *p* < 0.001). The cross tabulations of the outcome variable (positive or negative Tx effect on PSI) and the sum of coding are summarized in Table 4 and Figure 4. The more Components the interventions had, the more likely parenting stress was attenuated in the reviewed studies. This supports the importance of including all three Components to maximize potential efficacy of the interventions for parenting stress.

**TABLE 4:** The cross tabulations of the treatment effect on PSI and the sum of coding.

	Tx effect on PSI			
Sum of coding	Negative effect (Total # = 12)	Positive effect (Total # = 23)	Total # each row	% Positive effect
0	4	0	4	0%
1	5	1	6	16.67%
2	3	6	9	66.67%
3	0	16	16	100%

#### Relative contributions of three components to the Tx effects on parenting stress index

To explore relative contributions of three Components to the Tx effects on PSI, we examined the variance of the outcome variable (Tx effect on PSI) explained by the three Components, by performing logistic regression tests with different methods, namely enter, forward, and backward. In the enter model, all three Components were entered simultaneously as predictors, and they totally explained 79.8% of the total variance of the Tx effect on PSI, with 75% accuracy in predicting negative Tx effects (9 out of 12) and 95.7% accuracy predicting positive Tx effects (22 out of 23). In the forward model, Component 3 was selected to be the first single predictor that predicted the outcome the most, which explained 44.8% of the total variance, and subsequently Component 1 was added to the model, which additionally explained another 32.0% of the total variance, resulting in 76.8% of variance explained by Components 1 and 3. In the backward model, all three Components were initially included and subsequently Component 2 was removed from the model as its removal only reduced the total variance explained from 79.8 to 76.8%. Taken together, Components 1 and 3 were two relatively distinct predictors that explained 44.8 and 32% variances of the Tx effect on PSI, respectively, while Component 2 showed little added value in explaining the variance, which perhaps was due to its collinearity with Component 1. Together with correlation data presented in Table 2, these analyses based on logistic regression support the particular importance of Components 1 and 3 for developing effective parenting stress interventions in the future.

#### Summary of the theory-guided quantitative analysis

The theory-guided quantitative analysis of clinical studies of parenting interventions demonstrated the following points: (1) Evaluating parenting interventions on the basis of the three identified components motivated by the dyadic active inference model turned out to be useful and yielded consistent results in gaging the success or the failure of parenting interventions, and (2) the review’s findings seem to suggest the importance of including three identified therapeutic components to be implemented in the development of parenting interventions.

## Integrating our work with the literature on mother-infant dyadic interactions

To relate our work to the literature of developmental psychology succinctly, here we integrate the work that we presented above with the dyadic concepts that were identified in a systematic review of 82 unique studies on mother-infant dyadic processes, namely, Mutuality, Reciprocity, Attunement, Contingency, Coordination, Matching, Mirroring, Reparation, and Synchrony (Provenzi et al., 2018). While the authors of Provenzi et al. (2018) provided a theoretical description of the relationships among these dyadic concepts, they did not explicitly describe a formal model of a dyad in a strongly coupled state. We believe our work can complement the work of Provenzi et al. (2018), as discussed below.

In Table 5, Provenzi et al.’s theoretical definitions of the concepts of dyadic processes are listed in the second column; the relevance of these concepts to our dyadic model is described in the third column; the intervention components in our coding system that are likely to be involved in each of these dyadic concepts are listed in the fourth column.

**TABLE 5:** The relevance of present work to the literature of dyadic process as summarized in Provenzi et al. (2018).

Dyadic concepts	The concepts’ definition provided in Provenzi et al. (2018)	The concepts’ relevance to the dyadic active inference model in our work	The concepts’ relevance to the coding system of interventions
Mutuality	Mutual contribution of the interactive partners, which might not be equal in terms of frequency and intensity of the behaviors of the two partners.	The necessity of using a dyadic model to describe and understand person-person interactions in a strongly coupled state.	Component 1 (promotion of symbiotic benevolence)
Reciprocity	Reciprocal influence between interactive partners.	The interaction at the level of nodes S and A between two partners at one moment will produce an effect on each person’s internal model at the level of node M at the next moment after the interaction.	Component 1 (promotion of symbiotic benevolence)
Attunement	Sharing of actions and intentions which includes maternal identification of infant’s inner feelings/states and infant’s comprehension that the mother is referring to his own original state.	Attunement is very similar to intersubjectivity. As the internal modal (node M) of one partner is closely related to intentions that cause actions (node A) and feelings (node S) of the other partner subsequently causes internal model (node M), attunement is achieved when the mother’s M of infant is consistent with the infant’s M of his or her own nodes S and A.	Component 1 (promotion of symbiotic benevolence)
Contingency	Reciprocal adjustment of *trans*-modal affective and behavioral signals within a micro-temporal window that leads to infants’ learning and regulation skills and interactive patterns.	Contingency reflects the operational working of an active inference engine in which one’s internal model is optimized. The learning occurs after encountering surprisal and using perceptual inferences to minimize variational free energy. Skills are acquired after using active inference to minimize expected free energy.	Component 2 (intervention to reduce under-coupling)
Coordination	Bidirectional rhythmic exchanges characterized by specific timing and turn taking which facilitates the reciprocal prediction of future behavioral states.	Coordination is similar to the contingency in a strongly coupled state, wherein two persons take turn to observe, mirror, and respond to one another, creating rhythmic time-contingent dynamic relationships.	Components 2 (intervention to reduce under-coupling)
Matching	Simultaneous exhibition of the same affective and/or behavioral state by the mother and the infant.	Matching occurs in a strongly coupled state, wherein one person’s node A causes the other’s node S and *vice versa*. Because both persons’ behavioral states (node A’s) are similar, their affective states (nodes S’s) are also similar. Matching is like simultaneous mirroring that may be more automatic or spontaneous than intentional mirroring, below.	Components 2 (intervention to reduce under-coupling)
Mirroring	Exaggerated/marked reflection of *trans*-modal child behaviors by the mother through imitation of affective quality reproduction in a temporally contingent way.	Mirroring is a special form of matching when matching may be more deliberately or intentionally performed than simultaneous matching. Mirroring can happen bidirectionally.	Components 2 (intervention to reduce under-coupling)
Reparation	Dyadic process in which unmatched dyadic states are transformed in matched dyadic states producing an opportunity to learn interactive strategies and to achieve better stress and emotion regulation.	Reparation is the minimization of dyadic stress by using the surprisal or prediction errors in a dyadic interaction to update the internal model(s) to minimize the surprisal in the next interaction. Because stress is proportional to the surprise, the reduction of surprise can reduce stress.	Components 1 (promotion of symbiotic benevolence) and 2 (intervention to reduce under-coupling)
Synchrony	Degree of congruence between *trans*-modal behaviors of two partners which is lagged in time and which promotes infants’ learning of emotional regulation skills and the emergence of expectations on interactive repertoires.	Synchrony indexed by any observable indicators may reflect the degree of intersubjectivity as conceptualized in our dyadic model.	Components 1 (promotion of symbiotic benevolence) and 2 (intervention to reduce under-coupling)

The first two of the nine dyadic concepts identified in Provenzi et al. (2018) are Mutuality and Reciprocity, which are seen as overarching concepts underlying dyadic processes. In terms of our dyadic model, we consider Mutuality—mutual contribution of the interactive partners—as a concept to emphasize the strongly coupled state, as opposed to a weakly coupled state, between two persons. We consider Reciprocity—reciprocal influence between interactive partners—as a concept to emphasize the bi-directional cause and effect of the (A), (S), and (I) nodes of the dyadic active inference engine. Further, Provenzi et al. (2018) described two dynamic cycles emerging from their computer-aided text analysis, quoted in paragraphs below. ^**^

*“First, the ability to share intentions (i.e., attunement)—rather than simple behaviors or actions within the dyad—emerged as a more complex mutual engagement between the mother and the infant which is built upon low-level contingent engagement (i.e., contingency and coordination). From this perspective, mirroring should be considered as a specific way of being together, which might only appear when the mother is able to understand the behavioral and inner states of the infant in order to provide an exaggerated version of the observed and inferred infants’ socio-emotional state. When effective mirroring occurs, greater levels of contingency might be reached by the dyad, so that mother and infant constitute a dynamic system characterized by a behavioral-psychological self-organized and homeostatic cycle”* (Provenzi et al., 2018).

Apparently, the above description of the first cycle—which involves attunement, contingency, coordination, and mirroring—is compatible with our description of the dyadic active inference engines that are strongly coupled—that is, by virtue of the coupling between the two engines’ nodes (As) and (Ss) at the lower level, which result in the mirroring of the two agents, the attunement between the two engines’ (I) nodes are achieved at the higher level.

*“Second, a second cycle of matched and un-matched behavioral states within the dyad appeared to be regulated by dyadic reparation. Repeated matching emerged as the pre-condition for synchrony, which, in turn, contributed to heightened matching states. In other words, repeated in-moment matching states contribute to lagged moment-by-moment synchrony in time, so that reiterated interactive exchanges between mothers and infants grow in complexity in a reciprocal way”* (Provenzi et al., 2018).

Similarly, the above description of the second cycle—matching and the reparation of an unmatched state—is consistent with the process of perceptual and active inferences to update the prior beliefs such that the variational and expected free energy can be minimized eventually.

*“In sum,. coordination (of behaviors) and attunement (of intentions) might be considered as two critical nodes which allow the mother-infant dyad to move from behavioral forms of involvement (i.e., contingency, matching) to more complex psychological and inner-state forms of dyadic engagement (i.e., attunement, synchrony)”* (Provenzi et al., 2018).

In sum, the above description of coordination at the behavioral level and attunement at the intentional level is consistent with the two levels of an active inference engine, the behavioral coupling between the dyad’s nodes (As) and (Ss) in the lower level and the approximation of the dyad’s prior beliefs in nodes (Is) in the higher level, respectively, (see Figure 1).

In the fourth column of Table 5, we list the dyadic concepts’ relevance to the coding system of the interventions that we presented above. The intervention Components 1 and 2 aim to rehabilitate or promote the functioning that is largely relevant to the higher level, node (I), and lower level, nodes (A) and (S) of the active inference engines in a strongly coupled state, and both Components are similar to the concepts involved in the attunement of intentions and coordination of behaviors in Provenzi et al’s terms, respectively. However, none of the dyadic concepts identified in Provenzi et al. (2018) would seem relevant to Component 3 (intervention to reduce over-mentalizing) in our coding system. This is because Component 3 is not within the scope of the work undertaken by Provenzi et al. (2018) and, rather, it is by and large a therapeutic inner work on one’s awareness of self and other’s inner thoughts, feelings, and intentions to rectify the problem of over-mentalizing. To dig deeper in this issue, in the following sections, we turn to Buddhist Philosophy of Mind (which is synonymous to awareness in the present context) to discuss the relational worldview that may help explain the root cause of invalid beliefs, its resulting excessive dyadic stress and unnecessary suffering, and the innate capacity of awareness to be free from invalid beliefs and suffering.

In short, by proposing the causal relationship among deficient relational benevolence due to invalid beliefs, under-coupling, and over-mentalizing that would result in excessive stress and impaired intersubjectivity, we believe that our work complemented Provenzi et al.’s comprehensive review work by addressing the following issues: (a) how dyadic interactions can influence maternal wellbeing, which is acknowledged by Provenzi et al. (2018) as lacking in the current literature, (b) how the dyadic active inference model and hence the quality of dyadic interactions can be compromised, and (c) how to most effectively intervene therapeutically to counteract with compromised intersubjectivity, which is corroborated in our theory-guided quantitative analysis of the literature on parenting interventions and parenting stress.

## Understanding intersubjectivity and active inference in a relational worldview

While we have postulated that the hallmark of quality dyadic processes is intersubjectivity, how is intersubjectivity—the awareness of self and others—even possible in the first place? The answer depend on the “worldviews”—ontological and epistemological assumptions implicitly or explicitly used to understand any phenomena in this world—that are brought into the studies of awareness (mind) and/or metaphysics (mind-body relation; Avramides, 2020). While a worldview may not be easily falsifiable, not all worldviews garner scientific evidence equally. To make any science fruitful, the worldview that a scientific community adopts should be as consistent with the most fundamental nature of reality as possible. In this section, we discuss how a relational worldview supported by Physics and Buddhist Philosophy can enrich our understanding of the nature of active inference, awareness, and intersubjectivity.

### Toward an abstract expression of relational worldview according to physics and Buddhist Philosophy

As already described in the beginning, *living organisms are impermanently, inter-dependently self-organizing* through ceaseless interactions with their environments. It is emphasized in FEP that living organisms as active inference engines are self-evidencing in its environments by using their own actions to solicit their sensory inputs from the world. In a way, active inference is consistent with the notion of *participatory universe*—the outcome of measuring a quantum system depends on the apparatus chosen to perform the act of measuring—coined by John A. Wheeler, one of the greatest Physicists and Philosophers in our time.

Most physicists would agree that, ontologically, the universe is fundamentally relational, and, epistemologically, to be observable is to be interactable in physics (Rovelli, 2021). In the standard model of physics, fundamental particles are nothing but products of interactions of even more fundamental quantum fields (Peskin and Schroeder, 1995). The relational nature of the participatory universe has been rigorously demonstrated many times in a family of so-called *delayed-choice experiments*. This kind of experiments aims to demonstrate the dual nature of a quantum system, e.g., a single photon that can behave either like a particle or like a wave. In this kind of experiments, whether a single photon behaves particle-like or wave-like depends on whether a particle detector or wave detector is chosen to measure the photon’s behavior. Because the act of choosing either one of the detectors to observe the single photon occurred after the very photon has completed its behavior, this kind of experiments are thus called “delayed-choice.” After decades of rigorous delayed-choice experiments, most physicists would agree that:

“…*no elementary phenomenon is a phenomenon until it is a registered phenomenon*…*some registered phenomena do not have a meaning unless they are put in relationship with other registered phenomena*” (Ma et al., 2016).

The notion of participatory universe suggests that all information is relationally dependent upon the existence of observations or observers whereas the existence of observations or observers is relationally dependent upon the ingredients of the universe. The observer here may be a living system, which is modeled as an active inference engine, or simply a quantum-system measuring apparatus that solicits and thus co-creates the outcomes of observing an incoming event.

To summarize the relational worldview underlying active inference and participatory universe, we resort to the notion of “Dependent Origination” that can be abstractly expressed in the following equation:


(1) Effect=Cause×Condition

Colloquially, Eq. 1 should read “Effect is an interactive product of Cause by Condition.”

Both Cause and Condition are factors participating in an interaction that produces Effect. Among these factors, some are called “Cause,” if they maintain certain *systemic continuity* with the “Effect”; others are called “Condition,” if they lack apparent systemic continuity in relation to either Cause or Effect. The term *systemic continuity* is used here to refer to the relation between Cause and Effect—that they are continuous but successive phenotypes of the same system.

For example, fruit is an interactive product of its seed and other factors such as soil, bacteria, water, sunlight, farmer, etc. In this case, the fruit is Effect, the seed is Cause, and other factors are Conditions. There is systemic continuity between the seed (Cause) and fruit (Effect) because the seed and fruit belong to the same system defined by the same genes that they carry (systemic), but they never co-exist simultaneously in the temporal succession of seed and fruit (continuity). In contrast, while the other factors (soil, bacteria, water, sunlight, farmer, etc.) are necessary to produce the fruit, they are designated as Conditions because they lack the systemic continuity with either the seed or the fruit.

The interactive product, designated by the sign “×” in Eq. 1, renders Eq. 1 as a non-linear formula. Mathematically, the non-linearity mandates that Effect is neither a linear transformation of Cause, nor Condition, nor a linear combination thereof.

Interestingly, Eq. 1 can serve as a mathematical expression of the ultimate nature of reality, namely “Emptiness,” that has been established by Arya Nagarjuna, the founder of Madhyamaka School of Buddhist Philosophy, in the following reasoning:

“Neither from itself,

Nor from another,

Nor from both,

Nor without a cause,

Does anything whatever, anywhere arise.” (Nagarjuna, 1995) Ch. 1 V. 1

Arya Nagarjuna’s reasoning on Emptiness can be translated in terms of Eq. 1, as follows:

“Neither from self” means that the Effect is not a linear transformation of its Cause—, e.g., although the fruit and seed carry the same genes, the fruit is not identical to the seed or a scaled-up version of the seed.

“Nor from other” means that the Effect is not a linear transformation of its Condition—, e.g., the fruit is not identical to soil, bacteria, water, sunlight, farmer, etc., that do not even share the same genes with the fruit.

“Nor from both” means that the Effect is not a linear combination of the Cause and Condition—, e.g., the fruit is not just the sum of the seed, soil, bacteria, water, sunlight, farmer, etc. that do not have any interactions among them.

“Nor without a cause” means that the Effect is not something other than an interactive product of Cause by Condition—, e.g., the fruit does not come to exist without being the effect of the interactions among the seed, soil, bacteria, water, and other conditions.

“Does anything whatever, anywhere arise” means that nothing can be observed without following the ultimate nature of the participatory universe.

In short, Eq. 1 is not only an abstract expression of “Dependent Origination,” but also an axiomatic translation of Arya Nagarjuna’s reasoning on “Emptiness.” The resulting functional equivalence between “Emptiness” and “Dependent Origination” is eloquently reflected in the pith of Buddhist wisdom, as Je Tsongkhapa (1357–1419) stated in his masterpiece “In Praise of Dependent Origination” (Lobsang Gyatso and Woodhouse, 2011) that:

*“When one sees Emptiness in terms of the meaning of dependent origination*, *then being devoid of intrinsic existence and possessing valid functions do not contradict.”*

—Je Tsongkhapa, translated by Geshe Thupten Jinpa.

The consistency between the Buddhist wisdom and John A. Wheeler’s notion of participatory universe becomes evident when we summarize experimental evidence demonstrated in those delayed-choice experiments (Ma et al., 2016) as a special case of Eq. 1, that Effect of measuring a quantum system (e.g., a single photon) is an interactive product of Cause by Condition—wherein Cause is the to-be-measured single photon and Condition is the apparatus used to detect the photon’s particle-like or wave-like behavior. When Conditions favor the single photon’s wave-like or particle-like behavior, the photon will appear to behave like a wave or a particle, respectively, after it interacts with Conditions.

Altogether, the relational worldview in the current context specifically refers to the notion that Effect is as an interactive product of Cause by Condition, which can be equivalent to the notions of Dependent Origination and the ultimate nature of reality, Emptiness, in Buddhist Philosophy as well as the notion of Participatory Universe in Physics.

In the following sections, we will use similar abstract expressions to describe how intersubjectivity and active inference framework can be understood as additional special cases of this relational worldview.

### Understanding intersubjectivity in the relational worldview

*The acid test of every epistemology is, when all is said and done, the intersubjective relationship.*

(Fuchs, 2017, p. 27)

Intersubjectivity—the awareness of self and other’s intentions and feelings—is relational, because the effect of awareness in intersubjectivity depends on the interactive coupling between the participants. Here we apply the abstract expression of Dependent Origination to the nature of awareness and intersubjectivity.

According to the Buddhist science of mind, the nature of awareness is fundamentally relational, described as follows:

“The nature of cognition is stated to be awareness, and the nature of consciousness is said to be clear (or luminous) and aware. ‘Clear’ here expresses the essential nature of consciousness, and ‘aware’ expresses its function. ‘Clear’ also indicates: (1) that consciousness is beyond the nature of matter, which is characterized as tangible and obstructive, so it is clear in nature; (2) that just as reflections appear in a mirror, any internal or external object whatsoever—good or bad, pleasant or unpleasant—can appear in consciousness, so consciousness is luminous in that it illuminates objects; and (3) that the essential nature of consciousness is not contaminated by the stains of mental afflictions such as attachment, so its nature is clear or luminous.” (Tenzin Gyatso, 2020, p. 41).

The relational nature of the awareness is often likened to a clear lampshade or mirror metaphorically, as discussed in our previous work (Ho et al., 2021). In the former, a clear lampshade is colorless (clear) and any object that the mind perceives is like a light bulb in the clear lampshade, which can color the lampshade with its light, e.g., the lampshade’s color becomes blue when a light bulb emits blue light. However, just as the light bulb can never stain the lampshade, the object perceived by the mind can never stain the mind. Thus, the mind (lampshade) returns to its colorless clarity as soon as the object (light bulb) is turned off. In the latter, awareness is also likened to a mirror, as it reflects the object in front of the mirror, but the mirror is not the object nor the image in the mirror. In other words, the image in the mirror is an interactive product of the mirror and the object in front of the mirror.

Here we describe the mirror-like nature of the awareness in terms of the abstract expression of Dependent Origination. For an object, A, let a subject’s awareness of A be “A-ness,” which is called the “qualia” of perceiving A. As Effect is an interactive product of Cause by Condition, “A-ness” is an interactive product of subject (Cause) and objects (Conditions), which include the object A and other environmental conditions, e.g., the subject’s visual system and other physical environments. Note that the subject, not the object A, is designated as Cause because the qualia as Effect is a subjective experience that has the systemic continuity with the subjectivity of the subject, whereas the object A does not have such systemic continuity. Therefore, in the realm of awareness, Eq. 1 can be re-expressed as follows:


(2a) $${\text{Effect}}_{\left[\text{Qualia}\,`\,`\,\text{A}-\text{ness}\,"\right]}={\text{Cause}}_{\left[\text{Subject}\right]}\times {\mathrm{Condition}}_{\left[\mathrm{Object}\,A\right]}$$

Using the mirror metaphor of the mind, the qualia “A-ness” is like the image in the mirror (Effect), the subject’s mind is like the mirror (Cause), and the object A is like an object placed in front of a mirror (Condition).

In parallel, the object A should be changed after the subject-object interaction too. There should be a counterpart to Effect_[Qualia]_, which can be re-expressed as follows:


(2b) $${\text{Effect}}_{\left[\mathrm{Object}\,A^{\prime }\right]}={\text{Cause}}_{\left[\mathrm{Object}\,A\right]}\times {\mathrm{Condition}}_{\left[\text{Subject}\right]}$$

wherein Effect_[Object A’]_ denotes the post-interaction object A, with the object A as its Cause and the subjects of awareness and environmental objects (e.g., the brain system and lights in the room) as its Conditions. Due to the subject x object interaction, such Effect_[Object A’]_ is effectively infusing the object with certain “mental energy” in a process called “cathexis.” We will further discuss the concept of cathexis in the context of active inference framework below.

As the Ubuntu proverb says, “I am because you are,” there is no independent subject “I” that can be designated without also simultaneously designating the objects of “other” in relation to the “I.” The intersubjective awareness is a relational awareness of self and other. Trevarthen made a distinction between primary and secondary intersubjectivity (Trevarthen and Aitken, 2001). The former refers to “an infant’s active and immediately responsive conscious appreciation of the adult’s communicative intentions” and the latter refers to “the integration in the new form of cooperative person-person-object awareness” that combines object awareness (e.g., doing with things) and person awareness (e.g., communicating with persons).

The primary intersubjectivity is considered innate and mainly characterized by *embodied, affective, and intuitive forms of relationships*, preceding communications mediated by symbolic and verbal processes (Trevarthen and Aitken, 2001). A key attribute underlying primary intersubjectivity is spontaneous mimicry or voluntary imitation of others’ facial expressions or manual gestures. Infants show spontaneous facial mimicry soon after birth (Meltzoff and Moore, 1977). When primary intersubjectivity is successfully executed and maintained, it leads to establishing the sense of safety and assurance that everything is all right at that moment.

Secondary intersubjectivity, which emerges around 9 months of age, incorporates objects into the mother–infant interactions, forming a person-person-object triadic relationship (Trevarthen and Hubley, 1978). Jointly attending to an object of shared interest is seen as a critical step toward a mutual incorporation of the other’s perspective into shared experiences. The emergence of secondary intersubjectivity points to a major developmental milestone where shared joint activities between mother and infant can create a significantly more advanced level of interactive intersubjectivity that can support the development of higher cognitive capacity for language, reflection, and perspective-taking.

Here we focus on how to understand primary intersubjectivity in terms of the abstract expression of the relational worldview. Let Eqs 2a, 2b be applied to dyadic processes wherein two subjects, Person 1 (P1) and Person 2 (P2), are strongly coupled in their person-person interactions.

For Person 1,


(3a) $${\text{Effect}}_{\left[`\,`\,\mathrm{P1}\times \mathrm{P2}-\text{ness}\,"\,\mathrm{in}\,\mathrm{P1}\right]}={\text{Cause}}_{\left[\mathrm{P1}\right]}\times {\mathrm{Condition}}_{\left[\mathrm{P1}\times \mathrm{P2}\right]}$$

For Person 2,


(3b) $${\text{Effect}}_{\left[`\,`\,\mathrm{P2}\times \mathrm{P1}-\text{ness}\,"\,\mathrm{in}\,\mathrm{P2}\right]}={\text{Cause}}_{\left[\mathrm{P2}\right]}\times {\mathrm{Condition}}_{\left[\mathrm{P2}\times \mathrm{P1}\right]}$$

The notations, Effects, on the left side of the equations are: Effect_[“P1×P2–ness” in P1]_ denotes P1’s qualia about P1 × P2 dyadic interactions, i.e., “P1 × P2-ness,” and Effect_[“P2__×__P1–ness” in P2]_ denotes P2’s qualia about P2 × P1 dyadic interactions, i.e., “P2 × P1-ness.” The notations on the right side of the equations are: Cause_[P1]_ or Cause_[P2]_ denotes P1 or P2’s mirror-like awareness as the subjects; Condition_[P1__×__P2]_ or Condition_[P2__×__P1]_ refers to the conditions that interact with the mirror-like awareness, which can be any objects or behaviors of the dyadic system, e.g., the dyad’s brains, bodies, verbal or physical behaviors, during the dyadic interactions.

As mentioned above, the Buddhist notion of mirror-like awareness suggests that “the essential nature of consciousness is not contaminated by the stains of mental afflictions such as attachment, so its nature is clear or luminous.” (Tenzin Gyatso, 2020, p. 41). Eqs 3a, 3b do not guarantee that all qualia of “P1 × P2-ness,” or “P2 × P1-ness,” are equal in its level of intersubjectivity. Instead, the capacity of P1 or P2’s intersubjectivity (Effect_[“P1__×__P2–ness” in P1]_ or Effect_[“P2__×__P1–ness” in P2]_) depends on the quality of the conditions (Condition_[P1__×__P2]_ or Condition_[P2__×__P1]_) that one’s mirror-like awareness (Cause_[P1]_ or Cause_[P2]_) interacts with. In other words, Eqs 3a, 3b can be applied to any level of intersubjectivity, whether it is an optimal level of intersubjectivity (as depicted in Figure 2) or a sub-optimal level of impaired intersubjectivity (as depicted in Figure 3).

### Understanding active inference framework in the relational worldview

The relational worldview—Effect is an interactive product of Cause by Condition—can be applied to understand the active inference framework. In the active inference framework, actions in active inference co-create the perceptions with the incoming event, which is analogous to the facts that actions of measuring co-create the effects of the measurement of the quantum systems in delayed-choice experiments in Physics. When a person or agent is modeled as an active inference engine, the engine serves as the subject that interacts with an object in the external world. As an example, here we apply the abstract expression of Dependent Origination to the active inference process in the four-node network (Figure 1).

Let nodes (A), (S), and (I) of an active inference engine be the Cause and node (E) in the external state be objects or events be the Condition that interacts with the Cause in Eq. 1. Now we have a pair of expressions as follows:

For the effect on the active inference engine,


$${\text{Effect}}_{\left[\text{nodes}\,\left({\text{A}}^{\prime }\right),\,\left({\text{S}}^{\prime }\right),\,\left({\text{I}}^{\prime }\right)\right]}={\text{Cause}}_{\left[\text{nodes}\,\left(\text{A}\right),\,\left(\text{S}\right),\,\left(\text{I}\right)\right]}$$

(4a) $$\times {\mathrm{Condition}}_{\left[\text{node}\,\left(\text{E}\right)\right]}$$

wherein Effect_[nodes (A’), (S’), (I’)]_ designates the active inference engine *after* the Cause by Condition interaction, which involves iterations of perceptual and active inferences to optimize the prior beliefs to minimize the variational free energy in FEP. The systemic continuity between the Cause and Effect in Eq 4a is consistent with the notion that active inference engines are self-evidencing (Friston, 2018; Friston et al., 2022).

For the effect on the external state,


(4b) $${\text{Effect}}_{\left[\text{node}\,\left({\text{E}}^{\prime }\right)\right]}={\text{Cause}}_{\left[\text{node}\,\left(\text{E}\right)\right]}\times {\mathrm{Condition}}_{\left[\text{nodes}\,\left(\text{A}\right),\,\left(\text{S}\right),\,\left(\text{I}\right)\right]}$$

wherein Effect_[node__(E’)]_ designates the external state after the Cause by Condition interaction. Notably, the subject-by-object interaction effect on the object is denotated as the “cathexis” here, because it potentially entangles the object with certain orientation or propensity of the subject. For example, Edward Tolmen construed the process of cathexis as the learned tendency to associate certain objects with certain drives, which is one of the major determinants of choice behaviors, e.g., why meat lovers tend to satisfy their hunger with meat (positive cathexis) rather than non-meat products (negative cathexis; Tolman, 1945).

In the neuroscience literature, the cathexis effect is usually conceptualized in terms of the construct of incentive value—a positive or negative cathexis of an object is construed as a positive or negative incentive value of the object, respectively, (Dickinson and Balleine, 1994; Balleine and Dickinson, 1998). For example, a rat has tasted a very salty liquid solution and then does not consume the same solution anymore. In this case, the very salty solution has a negative incentive value for the rat.

The question is, after the negative incentive value of the solution has been established, what would the rat do when it is put in a salt-deprived state and then encounters the same salty solution again? This is the question ingeniously answered in an animal study, which showed that when the rat re-encountered the very salty solution after being put in a salt-deprived state surgically, it immediately ran toward and consumed the very salty solution appetitively (Robinson and Berridge, 2013).

As explained above, the non-linearity in those abstract expressions of Dependent Origination, including Eq. 4b, mandates the following three rules mathematically: The effect of Cause-by-Condition interaction, node (E’), is not identical to (1) any linear transformation of node (E), (2) nor a linear transformation of nodes (A), (S), (I), and (3) nor a linear combination of all four nodes. Hence, the incentive value of an object, *post-cathexis*, is not a property of the object, nor an internal representation encoded in the subject, nor a linear combination of the external object and internal representation.

The above axiomatic reasoning based on the non-linearity of Eq. 4b is corroborated by the experiments conducted by Robinson and Berridge (2013). As their study demonstrated an instant flip of the incentive value of the salty solution, from something negative to something positive, without any new learning, it is strongly suggested the following: The incentive value is not a property of the object, because the salty solution is not manipulated at all in their study. Nor the incentive value is encoded as the subject’s internal representation. The reason for this refutation is that, had this notion been true, the rat would have avoided the salty solution even when it was salt-deprived after surgery, because (a) presumably the internal representation of the salty solution in the brain was not altered by the experimental manipulations and (b) the rat did not have any new learning trials to encode a new internal representation of the solution’s incentive value in the novel salt-deprived state, as a representationalist account of the cathexis of incentive value would predict (Balleine and Dickinson, 1998).

This experimental refutation of the representationalist account of the cathexis of incentive value is potentially relevant to the refutation of the representationalist account of active inference, as noted in the FEP literature (Ramstead et al., 2020). The refutation of the representationalist accounts in the domains of incentive value and active inference speaks to the incompatibility between the relational worldview and realist worldview, to be further discussed below.

### The incompatibility between the relational worldview and realist worldview

As we abstractly denoted the relational worldview as the notion of Dependent Origination—Effect is an interactive product of Cause and Condition, a realist worldview presents a stark contrast. Realism can be defined as follows:

*“In general, where the distinctive objects of a subject-matter are a, b, c, and so on, and the distinctive properties are F-ness, G-ness, H-ness and so on, realism about that subject matter will typically take the form of a claim like the following: a, b, and c and so on exist, and the fact that they exist and have properties such as F-ness, G-ness, and H-ness is (apart from mundane empirical dependencies of the sort sometimes encountered in everyday life) independent of anyone’s beliefs, linguistic practices, conceptual schemes, and so on.”* (Miller, 2021)

In other words, the realism assumes a non-participatory universe wherein an object’s ultimate *real* ontology (*a, b, c and so on*) is *identical* to its properties (*F-ness, G-ness, H-ness and so on*) that can be observed and ascertained, independent of the subject/observer/apparatus involved in the observation. In an abstract expression, a realist worldview can be expressed as follows:


$${\text{Existence}}_{\left[a,\,b,\,c\,\mathrm{and}\,\mathrm{so}\,\mathrm{on}\right]}$$

(5) $$={\text{Observable}}_{\left[F-\text{ness},\,G-\text{ness},\,H-\mathrm{ness}\,\mathrm{and}\,\mathrm{so}\,\mathrm{on}\right]}$$

The incompatibility between the realist and relational worldviews is clearly evident in preceding discussions offered here. First, in contrast to the non-linearity in the abstract expression of relational worldview (Eqs 1–4), Eq. 5 is a linear function. Second, in contrast to Eqs 1–4, the subject or the apparatus used to make observation plays no roles at all in Eq. 5.

This incompatibility may point to the innate possibility of the cessation of suffering, i.e., healing. We postulated in Ho et al. (2021) that an invalid belief—the belief that self or other is fixed and unchanged after any person-to-person interactions—is the root cause of entrapping one’s awareness in an unhealthy state characterized by rigidity and inflexibility. The consequence of invalid beliefs in one’s awareness is that one will misattribute the cause of his or her subjective experiences (qualia) to the object itself, rather than the interactive product of his or her active inference engine by the object. In this misattribution, one judges the value of the object based on one’s own qualia (“this object makes me feel good/bad”), which one misbelieves to be identical to the object’s ultimate existence (“this object is good/bad in and of itself”), such that one would believe that he or she can only have or not have the object, rather than participate both in the creation of the qualia and in the unfolding of how the object appears to be in accordance with the ultimate nature of the reality, Dependent Origination.

In fact, invalid beliefs are deeply embedded in the realist worldview or beliefs. In Buddhism, such realist beliefs are called conceptual thoughts (*Vikalpas*), and the substantial bases underlying these realist beliefs are called mental fabrication or superimposition (*Prapañca*). In the root text of Madhyamaka Philosophy, Arya Nagarjuna provided the diagnosis and treatment for cyclic suffering caused by *Vikalpa and Prapañca*, stating:

“Action and misery having ceased, there is nirvana. Action and misery come from conceptual thought. This comes from mental fabrication. Fabrication ceases through emptiness.” (Nagarjuna, 1995) Ch. 18, V. 5

We have discussed the types of conceptual thoughts and levels of mental fabrication, as well as how these processes adversely influence the active inference processes of the brain in thinking about compassion elsewhere (Ho et al., 2021).

According to Buddhism, suffering is rooted in invalid beliefs and the cessation of suffering is guaranteed because all views laden with invalid beliefs are incompatible with the ultimate nature of reality (Tenzin Gyatso, 1997, 2009). Arya Nagarjuna’s prescription of cessation of suffering, as quoted above, can be paraphrased as follows: since the ultimate nature of reality—whether it is called Emptiness, Dependent Origination, or Participatory Universe in the context of the relational worldview—is incompatible with the realist worldview that can result in suffering, the cessation of suffering is always possible through the realization of the ultimate nature of reality by living in this participatory universe in the absence of invalid beliefs in one’s awareness and active inference engines.

Among the initial steps toward this goal, what we hoped to clarify through the abstract expressions presented above is the critical importance of cultivation of an inward, reflective contemplation, including being aware of one’s own prior beliefs in his or her internal states, the difference between the relational and realist worldviews underlying his or her prior beliefs, and the incompatibility between the realist worldview and the relational worldview concerning the nature of reality. Promoting such awareness is primarily an educational, contemplative work on changing beliefs and behaviors that are thought to hinder healthy dyadic interactions. By applying the abstract expressions and formal dyadic model to the study of the parenting interventions as an example domain, we indeed found that the cultivation of inward contemplation (i.e., Component 3 in the coding system) played a critical role in the efficacy of parenting interventions for reducing stress.

## Conclusion

*There is nothing more practical than a good theory.*

—Lewin (1952, p. 169)

*There is nothing as effective as the interdependence between theory, research, and practice.*

—David Bargal (Bargal, 2012)

In this hypothesis and theory paper, our work is presented with *interdependence* among the aspects of theory, research, and practice related to intersubjectivity. To recapitulate, these interdependent aspects include (1) intersubjectivity as a hallmark of quality dyadic processes; (2) a framework of active inference engine in weakly coupled and strongly coupled states; (3) how intersubjectivity can be impaired by deficient relational benevolence due to invalid beliefs, under-coupling, and over-mentalizing; (4) a theory-driven literature analysis to evaluate our hypotheses designed to determine the extent to which parenting interventions were effective in reducing parenting stress on the basis of our dyadic model; (5) how our work can be integrated with the literature of developmental dyadic processes; and (6) a series of abstract expressions/notations to elucidate the relational worldview as supported in multiple scientific domains, how the relational worldview differs from the realist worldview, and the importance of the awareness of such distinction in relieving oneself from suffering according to Madhyamaka Philosophy.

First, we used a well-established framework, namely Free-Energy Principle, to provisionally construe a dyadic active inference model of intersubjectivity. Specifically, two persons, as active inference engines, are strongly coupled when one person’s action causes the other’s feeling and *vice versa*. In a strongly coupled dyadic system, variational free energy will be collectively minimized in a state of high-level intersubjectivity. The literature has suggested that stress can be defined as excessive variational free energy that threatens a person’s self-centered beliefs. Thus, the dyadic active inference model predicts that a high level of intersubjectivity in a strongly coupled dyad will lead to minimize variational free energy and stress, while a low level of intersubjectivity in an under-coupled dyad will lead to engender excessive variational free energy and stress. Using child and mother as a focal example of person-to-person interactions in our investigation of intersubjectivity, our provisional work has led to identify three inter-related components to predict the compromised levels of intersubjectivity and increased stress, and we suggested three relational components and underlying brain networks that can serve as potential treatment targets for parenting interventions to reduce parenting stress.

Second, the results from quantitative evaluation of reviewed studies suggest that (1) the presence of any one of the three components was associated with success of parenting stress interventions and (2) the more components were included in an intervention, the more likely it was effective in reducing parenting stress. Pragmatically speaking, future intervention programs designed to attenuate parenting stress, regardless of any specific clinical therapeutic orientation, should consider the implementation of the three components to reduce parenting stress by enhancing the level of intersubjectivity in parent-child dyads.

Third, we integrated our work with decades of research in developmental psychology by comparing our work with the dyadic concepts identified in a recent systematic comprehensive review of dyadic processes (Provenzi et al., 2018). The compatibility between our dyadic model and the conceptual framework provided in Provenzi et al. (2018), along with the success in the theory-driven literature analysis in clinical studies, strongly supported the usefulness of our dyadic model in advancing the study of dyadic interactive process for future clinical application.

Fourth, using abstract expressions of Dependent Origination—Effect is an interactive product of Cause by Condition—in multiple domains, we explored the normality, impairment, and rehabilitation of intersubjectivity through the lens of the relational worldview.

In short, we presented an overarching framework grounded in the relational worldview for understanding the nature of reality. Articulating the relational worldview as effectively as possible may be the key to unlock the Team Human’s potentials to overcome human-made problems. While we desperately need to rebuild a viable Team Human to respond to multiple planetary challenges (wars, violence, climate change, poverty, erosion of trust, collapse of democracy, etc.), we suggest that the rehabilitation of intersubjectivity should take a center stage in our collective effort to mitigate harms that are caused by humans.

## Data availability statement

The original contributions presented in this study are included in the article/supplementary material; further inquiries can be directed to the corresponding author.

## Author contributions

SH is the principal developer of the theoretical framework and hypotheses and writer of the manuscript, he created the figures in the study. YN has collaborated with SH in developing and refining the theoretical framework, he also co-wrote the manuscript. MG contributed to quantitative analysis of parenting intervention studies, especially in reviewing and coding of those studies. JS co-wrote the manuscript and supported the research involved in the manuscript. All authors contributed to the article and approved the submitted version.
